# Microbial dysbiosis as a diagnostic marker in psychiatric disorders: a systematic review of gut–brain axis disruptions

**DOI:** 10.3389/fnins.2026.1728473

**Published:** 2026-02-03

**Authors:** Paula Espinosa, Mario S. Hinojosa-Figueroa, Paula Vallejo, Felipe Pérez, Gabriela Burneo, Coralía Villarreal, Jose A. Rodas, Jose E. Leon-Rojas

**Affiliations:** 1 NeurALL Research Group, Quito, Ecuador; 2 Escuela de Medicina, Universidad Internacional del Ecuador, Quito, Ecuador; 3 Cerebro, Emoción, Conducta (CEC) Research Group, Escuela de Medicina, Universidad de las Américas (UDLA), Quito, Ecuador; 4 School of Psychology, University College Dublin, Dublin, Ireland; 5 Escuela de Psicología, Universidad Espíritu Santo, Samborondón, Ecuador; 6 Grupo de Investigación Bienestar, Salud y Sociedad, Escuela de Psicología y Educación, Universidad de Las América, Quito, Ecuador

**Keywords:** diagnostic biomarkers, dysbiosis, gut microbiome, microbiome-gut–brain axis, psychiatric disorders, systematic review

## Abstract

**Background/objectives:**

Mental health disorders represent a major global health burden. Recent interest has surged in the microbiome–gut–brain axis, which may influence psychiatric pathophysiology. This systematic review evaluates alterations in intestinal microbiome (IM) composition between individuals with psychiatric disorders—such as schizophrenia, autism spectrum disorder (ASD), mood and eating disorders—and healthy controls, with a focus on diagnostic relevance.

**Methods:**

We conducted a systematic review across PubMed, Scopus, CENTRAL, and PsycINFO, following PRISMA 2020 guidelines. Studies were included if psychiatric diagnoses were made using DSM-V and intestinal dysbiosis was characterised at the phylum, family, and genus levels. Only observational and interventional studies were considered. Microbial alterations were extracted and analysed both qualitatively and quantitatively. Risk of bias was assessed using NIH Quality Assessment Tools.

**Results:**

A total of 80 studies involving 2,691 participants met the inclusion criteria. Across disorders, consistent disruptions were observed in *Firmicutes*, *Bacteroidetes*, and *Actinobacteria* phyla presented as the percentage of affected patients within each disorder. Autism spectrum disorder (ASD) was associated with decreased *Firmicutes* (↓ 4.79%) and *Bacteroidetes* (↓ 3.29%) and increased *Bifidobacteria*ceae (↑ 5.86%) and *Eggerthellaceae* (↑ 5.50%). Mood disorders, including major depressive disorder and bipolar disorder, showed increased *Christensenellaceae* (↑ 18.1%) and decreased *Ruminococcaceae* (↓ 2.0%). Schizophrenia was marked by elevations in *Lachnospiraceae*, *Christensenellaceae*, and *Enterobacteriaceae* (↑ 11–28%) and reductions in *Akkermansia* and *Turicibacteraceae* (↓ 9–28%). Anorexia nervosa and binge eating disorder displayed profound dysbiosis, including ↓ *Lactobacillus* (48.5%) and complete loss of *Akkermansia* (100%). ADHD showed a *Firmicutes*/*Bacteroidetes* imbalance (↑ 49.8%, ↓ 56.6%). These alterations suggest microbial signatures that are both disorder-specific and partially overlapping.

**Conclusion:**

Our findings highlight reproducible patterns of gut microbial dysbiosis that may represent candidate microbial biomarkers and inform future diagnostic research. Microbiome profiling has potential as a non-invasive adjunct to psychiatric diagnosis, warranting further exploration. Future longitudinal and mechanistic studies using standardised methods are essential to validate these microbial signatures and their diagnostic utility.

**Systematic review registration:**

https://www.crd.york.ac.uk/PROSPERO/view/CRD42021254293, CRD42021254293.

## 1 Introduction

A crucial, and often neglected, aspect of a human’s wellbeing and thriving is mental health. When the quality of life of an individual is affected by a significant impairment in behavior, emotional regulation, or cognition, mental health or psychiatric disorders arise. Approximately 13% of the global disease burden is attributed to psychiatric diseases, which are among the most challenging conditions to manage; worldwide, in 2019, 293 million between 5 to 24 years of age had at least one mental disorder, representing a mean prevalence of 11.63 (Kieling et al., 2024). Furthermore, it has been estimated that, in 2019, mental disorders caused a total loss of 418 million disability-adjusted life years (DALYs) and represented an economic burden of approximately USD 5 trillion (Arias et al., 2022). Environmental and individual factors interact and affect a person’s mental health across all life stages. Environmental elements encompass social, economic, and geopolitical conditions, whereas individual aspects pertain to heredity, lifestyle, diet, and emotional competencies (World Health Organization, 2025). Numerous protective and risk factors for the onset of psychiatric disorders have been recognised; yet, a definitive psychopathological explanation for these factors remains elusive (World Health Organization, 2025).

In recent years, there has been growing interest in the interplay between the intestinal microbiome (IM) and its effect on brain processes and behavior. Certainly, the bacteria that inhabit the human intestine may have a significant role not only in gastrointestinal health but also in the psychoneurological stability of its host (Berding et al., 2021). The bidirectional communication between the brain and the IM has been defined as the microbiome-gut-brain axis (Berding et al., 2021). The diverse composition of the IM includes bacteria, fungi, viruses, and certain protists (Berding et al., 2021; Góralczyk-Bińkowska et al., 2022). The IM fulfils several functions, such as the digestion and absorption of food, toxin neutralization, promotion of healthy intestinal peristalsis, and the creation of an intestinal barrier. All these functions and the communication between the IM and the nervous system are possible via metabolic, endocrine, neural, and immunological pathways (Góralczyk-Bińkowska et al., 2022). Specifically, this brain-gut connection begins as early as intrauterine life and is afterwards influenced by multiple factors such as the birth mode (c-section or vaginal delivery), diet, lifestyle, psychological stress, environmental exposure, and even the individual’s circadian rhythm (Góralczyk-Bińkowska et al., 2022). The various methods to promote a healthy IM have rendered it a focal topic of scientific inquiry, with the anticipation that understanding the components of a healthy IM may enhance mental health promotion.

There is an increasing amount of evidence suggesting that the IM composition and functionality vary between healthy individuals and the ones affected by multiple illnesses, including psychiatric disorders. Nonetheless, the influence of the IM composition and its significance in psychiatric diseases remain inadequately investigated (Afroz and Manchia, 2023). Therefore, this systematic review aims to comprehensively investigate qualitative and quantitative alterations in the intestinal microbiome of individuals with psychiatric disorders compared with healthy controls, with the dual objective of advancing understanding of microbiome-related psychopathological mechanisms and evaluating the potential diagnostic relevance of disorder-specific microbial signatures.

## 2 Materials and methods

Our review follows the recommendations of the Preferred Reporting Items for Systematic reviews and Meta-Analyses (PRISMA) 2020 guidelines and its protocol has been registered in PROSPERO (CRD42021254293).

### 2.1 Eligibility criteria

Our inclusion criteria were articles that investigated the gut microbiome and the effects on the development and/or prognosis of psychiatric disorders in patients diagnosed by a healthcare professional using DSM-V and DSM-IV criteria, as well as scales approved by the DSM. Only primary studies (cross-sectional, cohort, case–control, case series, and clinical trials) in both English and Spanish were included in this systematic review. There were no limitations related to the year of publication, age, gender, or ethnicity of the participants. The exclusion criteria included neuropsychiatric disorders caused by an identifiable organic lesion (i.e., tumour, ischaemia, etc.); animal and in-vitro studies were also excluded. Studies that considered self-reporting or the use of a diagnostic tool other than the DSM-V or those approved by the DSM-V, were not considered.

### 2.2 Information sources and search strategy

We queried the most relevant biomedical databases for our study focus, including MEDLINE (PubMed), Scopus, CENTRAL, and PsycINFO until February 13, 2022. Searches were supplemented by manual retrieval of any additional articles meeting eligibility criteria found in the references of selected articles. The key terms used in the search were gastrointestinal microbiome, mental disorders, psychiatric disorders, microbiome-gut–brain axis, gut–brain axis, neuropathogenesis, immune system, dysbiosis, and their variants; the complete search strategy used in each database can be found in the Supplementary materials.

### 2.3 Selection process

Two blinded authors independently reviewed the titles and abstracts of all the articles, after deduplication, against the aforementioned eligibility criteria; if any discrepancies were identified, a third author weighed in until mutual consensus was achieved. Afterwards, in a similar fashion, the remaining articles were further assessed by reading the full-text. Articles that successfully passed the process were then scrutinized for relevant information that was recollected into an Excel spreadsheet. Studies using ICD-10 or ICD-11 diagnostic criteria were included, as these frameworks are internationally harmonized with DSM classifications for the psychiatric disorders considered. In addition, disorder-specific validated diagnostic instruments (e.g., ADOS, ADI-R, K-SADS) were included when they operationalize DSM- or ICD-based diagnoses. Studies relying solely on self-report measures or non-validated screening tools were excluded. No analytical weighting was applied based on diagnostic framework, and all included studies were synthesized descriptively.

### 2.4 Data collection process and data items

Data was collected individually by the reviewers and any discrepancy was solved by discussion and mutual consensus. Data was extracted in an excel spreadsheet that contained the following variables: article identifying information (i.e., DOI, authors, year), sample size, study design, number, gender, and age of participants, prior psychiatric diagnosis, tool used for diagnosis, microbiome assessment, bacteria identified and categorized by phylum, family, and genus, change in the bacteria identified, and correlations with the psychiatric condition.

### 2.5 Risk of bias assessment

We assessed risk of bias in the included studies using NIH’s Study Quality Assessment Tools—namely the Quality Assessment of Controlled Intervention Studies Tool, the Quality Assessment Tool for Observational Cohort and Cross-Sectional Studies, the Quality Assessment of Case–Control Studies tool, and the Quality Assessment Tool for Case Series Studies—for evaluation of experimental, cohort, cross-sectional, case–control, and case series studies, respectively (NHLBI and NIH, 2025). Two authors independently applied the respective tool to each included study and recorded the answers for every question. There were three possible answers: yes, no, or other (cannot determine or not applicable). We then calculated a percentage for every study based on the number of yes out of the total number of questions. We classified every study into three categories: minimally low risk if the percentage of “yes” was 80% or higher, moderately low risk if the percentage was between 50 and 79%, and high risk if the percentage was less than 50%. Any disagreements were resolved by a third author who used the same methodology.

### 2.6 Effect measures and synthesis methods

To quantify the effect measure we calculated the relationship between the sample size and the change proportion in the microbiota for each mental disorder, considering the total amount of participants within each category of mental disorder diagnosis, and the bacteria presented in each case. We obtained an increase or decrease percentage value for each bacterium. The distribution of mental disorder categories was made as follows: (1) Schizophrenia; (2) autism spectrum disorder; (3) mood disorders including major depressive disorder (MDD), generalized anxiety disorder (GAD), and bipolar disorder (BPD); (4) attention deficit hyperactivity disorder (ADHD); (5) eating disorders including anorexia nervosa and binge eating disorder; and, (6) other mental disorders such as hypoactive sexual disorder, sleep disorders [idiopathic rapid eye movement (REM) sleep behavior disorder (iRBD)] and post-traumatic stress disorder (PTSD).

All reported outcomes were arranged in tables that highlighted the detailed differences in the gut microbiome at the phylum, family, and genus levels between the case subjects and the control groups. The results from each study were summarized by categorizing changes in the relative abundance (percentage), absolute abundance (counts), or diversity of each microorganism as increased, decreased, or unchanged. To summarize microbiome alterations across heterogeneous studies, we calculated descriptive percentages reflecting the proportion of participants within each psychiatric disorder category for whom a given taxon was reported as increased or decreased relative to controls. These values were derived by aggregating participant counts from individual studies reporting a directional change in a specific taxon and expressing this number as a percentage of the total number of participants within that diagnostic category. Importantly, these percentages do not represent pooled prevalence estimates, effect sizes, or meta-analytic measures, nor do they reflect the proportion of studies reporting a given change. Rather, they provide a descriptive synthesis intended to illustrate the relative consistency and recurrence of reported microbial alterations across the included literature. Accordingly, these percentages should not be interpreted as true prevalence rates or measures of association strength.

## 3 Results

### 3.1 Study selection and characteristics

**Figure 1:**
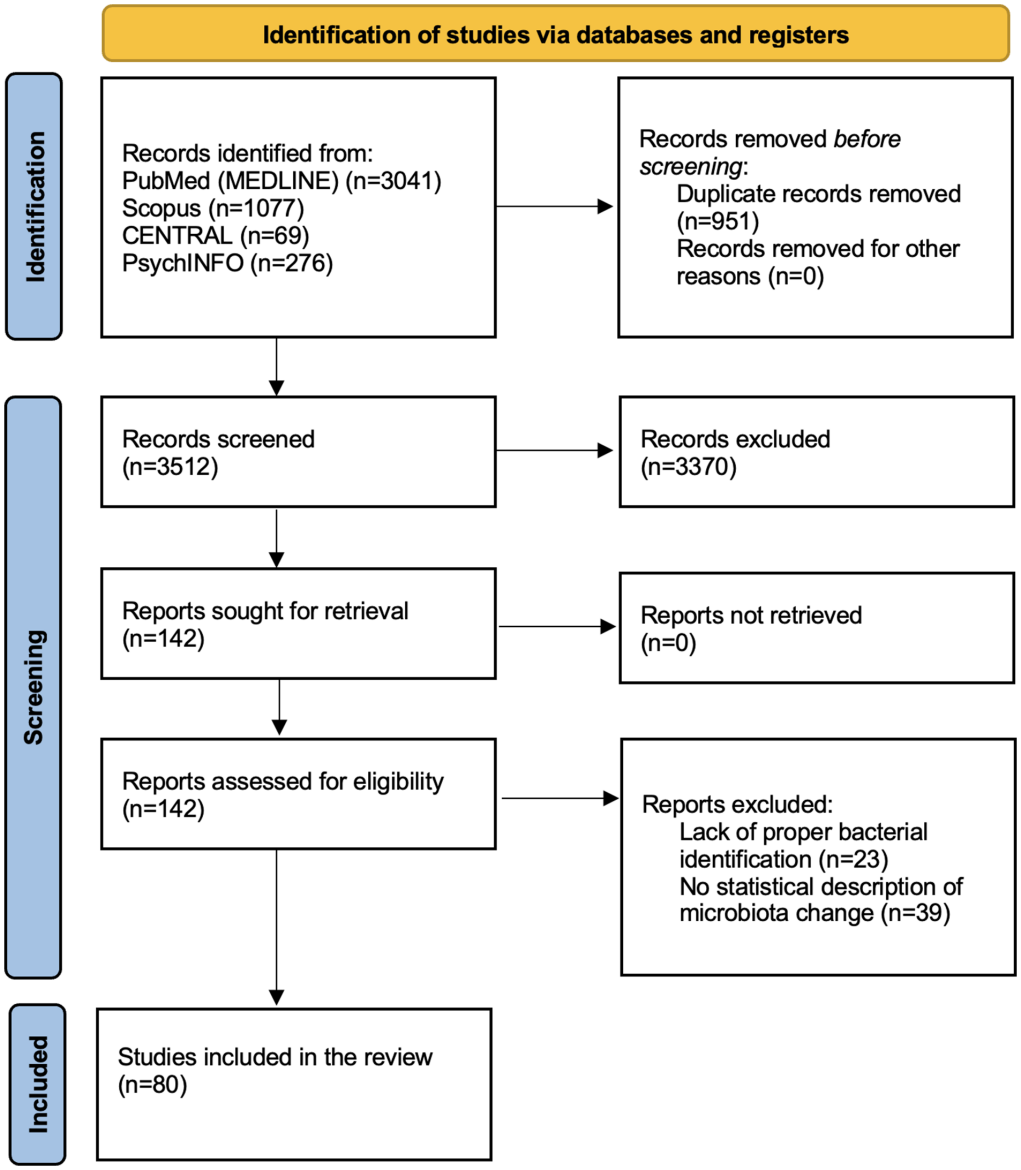
PRISMA 2020 flowchart showcasing the article selection process.

Our search yielded a total of 3,512 articles, after duplicate removal, of which 80 articles were finally selected for our review, providing a total of 2,691 participants (Aarts et al., 2017; Aizawa et al., 2016; Armougom et al., 2009; Averina et al., 2020; Bojović et al., 2020; Borgo et al., 2017; Cao et al., 2021; Carissimi et al., 2019; Chen et al., 2018, 2019, 2020; Chung et al., 2019; Coello et al., 2021; Angelis et al., 2013; Dong et al., 2020; Evans et al., 2017; Finegold et al., 2010; Gondalia et al., 2012; Grimaldi et al., 2018; Hanachi et al., 2019; Hemmings et al., 2017; Hua et al., 2020; Huang et al., 2018; Inoue et al., 2016; Pärtty et al., 2015; Jiang et al., 2015; Jiang H. -Y. et al., 2018; Jiang H. et al., 2018; Kang et al., 2013, 2017, 2018, 2019; Kleiman et al., 2015; Kong et al., 2019; Kushak et al., 2017; Lai et al., 2021; Leyrolle et al., 2021; Li et al., 2019, 2020, 2021; Lin et al., 2017; Liu B. et al., 2019; Liu et al., 2017; Liu S. et al., 2019; Lu et al., 2019; Luna et al., 2017; Ma et al., 2019, 2020; Mason et al., 2020; Morita et al., 2015; Naseribafrouei et al., 2014; Nguyen et al., 2019, 2021; Nishiwaki et al., 2020a; Painold et al., 2019; Pan et al., 2020; Pełka-Wysiecka et al., 2019; Plaza-Díaz et al., 2019; Prehn-Kristensen et al., 2018; Rong et al., 2019; Shaaban et al., 2018; Shen et al., 2018; Son et al., 2015; Strati et al., 2017; Sun et al., 2019; Tomova et al., 2020; Vinberg et al., 2019; Wan et al., 2020; Wang et al., 2019a,b; Williams et al., 2011, 2012; Yuan et al., 2018; Zhai et al., 2019; Zhang et al., 2018; Zhang M. et al., 2020; Zhang X. et al., 2020; Zou et al., 2021; Zurita et al., 2020; Pulikkan et al., 2018). The complete screening process can be found on Figure 1. Our review considered a variety of diagnoses including psychotic disorders (schizophrenia and first psychotic episode), autism spectrum disorder, mood disorders (major depressive disorder and bipolar disorder), generalized anxiety disorder, eating disorders (anorexia nervosa, food addiction), post-traumatic stress disorder (PTSD), and sleep disorders.

The diagnostic tools used across the studies included the Diagnostic and Statistical Manual of Mental Disorders (DSM), fifth and fourth editions (DSM-IV, DSM-5), International Classification of Diseases (ICD), tenth and eleventh revisions (ICD-10 and ICD-11), Autism Diagnostic Interview–Revised (ADI-R), Autism Diagnostic Observation Schedule (ADOS), ADOS-2, Kiddie Schedule for Affective Disorders and Schizophrenia (K-SADS), the Hamilton Depression Rating Scale, Autism Treatment Evaluation Checklist (ATEC), Pervasive Developmental Disorder Behavior Inventory (PDD-BI), and the Classification of Sleep Disorders Criteria-Third Edition (Aarts et al., 2017; Aizawa et al., 2016; Armougom et al., 2009; Averina et al., 2020; Bojović et al., 2020; Borgo et al., 2017; Cao et al., 2021; Carissimi et al., 2019; Chen et al., 2018, 2019, 2020; Chung et al., 2019; Coello et al., 2021; Angelis et al., 2013; Dong et al., 2020; Evans et al., 2017; Finegold et al., 2010; Gondalia et al., 2012; Grimaldi et al., 2018; Hanachi et al., 2019; Hemmings et al., 2017; Hua et al., 2020; Huang et al., 2018; Inoue et al., 2016; Pärtty et al., 2015; Jiang et al., 2015; Jiang H. -Y. et al., 2018; Jiang H. et al., 2018; Kang et al., 2013, 2017, 2018, 2019; Kleiman et al., 2015; Kong et al., 2019; Kushak et al., 2017; Lai et al., 2021; Leyrolle et al., 2021; Li et al., 2019, 2020, 2021; Lin et al., 2017; Liu B. et al., 2019; Liu et al., 2017; Liu S. et al., 2019; Lu et al., 2019; Luna et al., 2017; Ma et al., 2019, 2020; Mason et al., 2020; Morita et al., 2015; Naseribafrouei et al., 2014; Nguyen et al., 2019, 2021; Nishiwaki et al., 2020a; Painold et al., 2019; Pan et al., 2020; Pełka-Wysiecka et al., 2019; Plaza-Díaz et al., 2019; Prehn-Kristensen et al., 2018; Rong et al., 2019; Shaaban et al., 2018; Shen et al., 2018; Son et al., 2015; Strati et al., 2017; Sun et al., 2019; Tomova et al., 2020; Vinberg et al., 2019; Wan et al., 2020; Wang et al., 2019a,b; Williams et al., 2011, 2012; Yuan et al., 2018; Zhai et al., 2019; Zhang et al., 2018; Zhang M. et al., 2020; Zhang X. et al., 2020; Zou et al., 2021; Zurita et al., 2020; Pulikkan et al., 2018).

### 3.2 Risk of bias

Table 1 showcases the calculated risk of bias, and related scores, that resulted from applying the NIH’s Study Quality Assessment Tools (NHLBI and NIH, 2025).

**Table 1:** Risk of bias assessment.

Author (year)	Study design	N° Yes	N° No	N° N/A	% Yes	Level of bias
Aarts et al. (2017)	Cross-sectional	7	4	2	54%	Moderately low risk
Aizawa et al. (2016)	Cross-sectional	6	3	5	43%	High risk
Armougom et al. (2009)	Cross-sectional	8	4	2	57%	Moderately low risk
Averina et al. (2020)	Cross-sectional	7	4	3	50%	Moderately low risk
Bojović et al. (2020)	Cross-sectional	8	4	2	57%	Moderately low risk
Borgo et al. (2017)	Cross-sectional	6	4	2	50%	Moderately low risk
Cao et al. (2021)	Cross-sectional	6	6	2	50%	Moderately low risk
Carissimi et al. (2019)	Cross-sectional	6	4	2	50%	Moderately low risk
Chen et al. (2020)	Cross-sectional	7	4	3	50%	Moderately low risk
Chen et al. (2018)	Cross-sectional	6	3	5	43%	High risk
Chen et al. (2019)	Cross-sectional	8	3	3	57%	Moderately low risk
Chung et al. (2019)	Case control	9	2	1	75%	Moderately low risk
Coello et al. (2021)	Cross-sectional	7	5	2	50%	Moderately low risk
Angelis et al. (2013)	Cross-sectional	6	4	2	50%	Moderately low risk
Dong et al. (2020)	Cross-sectional	6	3	5	43%	High risk
Evans et al. (2017)	Cross-sectional	8	4	2	57%	Moderately low risk
Finegold et al. (2010)	Cross-sectional	7	4	1	58%	Moderately low risk
Gondalia et al. (2012)	Cross-sectional	7	4	1	58%	Moderately low risk
Grimaldi et al. (2018)	Clinical trial	11	1	2	79%	Moderately low risk
Hanachi et al. (2019)	Case control	8	4	2	57%	Moderately low risk
Hemmings et al. (2017)	Cross-sectional	7	4	3	50%	Moderately low risk
Hua et al. (2020)	Cross-sectional	7	4	3	50%	Moderately low risk
Huang et al. (2018)	Cross-sectional	6	3	5	43%	High risk
Inoue et al. (2016)	Cross-sectional	6	4	4	43%	High risk
Pärtty et al. (2015)	Clinical trial	12	1	1	86%	Minimally low risk
Jiang et al. (2015)	Cross-sectional	7	4	3	50%	Moderately low risk
Jiang H. -Y. et al. (2018)	Cross-sectional	10	3	1	71%	Moderately low risk
Jiang H. et al. (2018)	Cross-sectional	6	4	4	43%	High risk
Kang et al. (2013)	Cross-sectional	6	7	1	43%	High risk
Kang et al. (2017)	Cohort	9	4	1	64%	Moderately low risk
Kang et al. (2018)	Cross-sectional	5	5	4	36%	High risk
Kang et al. (2019)	Cohort	9	4	1	64%	Moderately low risk
Kleiman et al. (2015)	Cohort	8	3	3	57%	Moderately low risk
Kong et al. (2019)	Cross-sectional	7	4	1	58%	Moderately low risk
Kushak et al. (2017)	Cross-sectional	7	4	1	58%	Moderately low risk
Lai et al. (2021)	Cross-sectional	7	4	3	50%	Moderately low risk
Leyrolle et al. (2021)	Cross-sectional	8	3	3	57%	Moderately low risk
Li et al. (2021)	Case control	7	4	1	58%	Moderately low risk
Li et al. (2019)	Cross-sectional	7	4	3	50%	Moderately low risk
Li et al. (2020)	Cross-sectional	7	4	3	50%	Moderately low risk
Lin et al. (2017)	Cross-sectional	6	4	4	43%	High risk
Liu B. et al. (2019)	Case control	7	4	1	58%	Moderately low risk
Liu et al. (2017)	Clinical trial	9	3	0	75%	Moderately low risk
Liu S. et al. (2019)	Cross-sectional	8	3	3	57%	Moderately low risk
Lu et al. (2019)	Clinical trial	8	6	0	57%	Moderately low risk
Luna et al. (2017)	Cross-sectional	8	4	2	57%	Moderately low risk
Ma et al. (2019)	Cross-sectional	9	5	1	60%	Moderately low risk
Ma et al. (2020)	Cross-sectional	8	5	1	57%	Moderately low risk
Mason et al. (2020)	Cross-sectional	8	4	2	57%	Moderately low risk
Morita et al. (2015)	Cross-sectional	8	3	3	57%	Moderately low risk
Naseribafrouei et al. (2014)	Cross-sectional	9	3	2	64%	Moderately low risk
Nguyen et al. (2019)	Cross-sectional	8	3	3	57%	Moderately low risk
Nguyen et al. (2021)	Cross-sectional	8	3	3	57%	Moderately low risk
Nishiwaki et al. (2020a)	Cross-sectional	9	2	3	64%	Moderately low risk
Painold et al. (2019)	Cross-sectional	8	3	3	57%	Moderately low risk
Pan et al. (2020)	Cross-sectional	8	3	3	57%	Moderately low risk
Pełka-Wysiecka et al. (2019)	Cohort	9	5	0	71%	Moderately low risk
Plaza-Díaz et al. (2019)	Cross-sectional	8	3	3	57%	Moderately low risk
Prehn-Kristensen et al. (2018)	Cross-sectional	8	3	3	57%	Moderately low risk
Pulikkan et al. (2018)	Cross-sectional	9	2	3	64%	Moderately low risk
Rong et al. (2019)	Cross-sectional	8	3	3	57%	Moderately low risk
Shaaban et al. (2018)	Clinical trial	10	4	0	71%	Moderately low risk
Shen et al. (2018)	Cross-sectional	8	3	3	57%	Moderately low risk
Son et al. (2015)	Cross-sectional	8	3	3	57%	Moderately low risk
Strati et al. (2017)	Cross-sectional	8	3	3	57%	Moderately low risk
Sun et al. (2019)	Cross-sectional	8	3	3	57%	Moderately low risk
Tomova et al. (2020)	Cross-sectional	8	3	3	57%	Moderately low risk
Vinberg et al. (2019)	Cross-sectional	9	3	2	64%	Moderately low risk
Wan et al. (2020)	Case control	9	2	1	75%	Moderately low risk
Wang et al. (2019a)	Cross-sectional	8	3	3	57%	Moderately low risk
Wang et al. (2019b)	Cross-sectional	8	3	3	57%	Moderately low risk
Williams et al. (2011)	Cross-sectional	8	3	3	57%	Moderately low risk
Williams et al. (2012)	Cross-sectional	8	3	3	57%	Moderately low risk
Yuan et al. (2018)	Clinical trial	7	7	0	50%	Moderately low risk
Zhai et al. (2019)	Cross-sectional	8	3	3	57%	Moderately low risk
Zhang et al. (2018)	Cross-sectional	8	3	3	57%	Moderately low risk
Zhang M. et al. (2020)	Cross-sectional	9	2	3	64%	Moderately low risk
Zhang X. et al. (2020)	Cross-sectional	8	3	3	57%	Moderately low risk
Zou et al. (2021)	Cross-sectional	8	3	3	57%	Moderately low risk
Zurita et al. (2020)	Case control	9	2	1	75%	Moderately low risk

Overall, the methodological quality of the included studies was moderate. Although most studies met criteria for classification as having a moderately low risk of bias according to NIH assessment tools, many scores clustered near the lower bound of this category (approximately 50–60%), indicating relevant methodological limitations. A smaller subset of studies was classified as high risk of bias (Aizawa et al., 2016; Chen et al., 2018; Dong et al., 2020; Huang et al., 2018; Inoue et al., 2016; Jiang H. et al., 2018; Kang et al., 2013, 2018; Lin et al., 2017) and only study was considered to have minimally low risk of bias (Pärtty et al., 2015). These findings suggest that the current evidence base should be interpreted with caution.

### 3.3 Changes of intestinal microbiome (IM) in psychiatric disease and diagnostic potential

**Figure 2:**
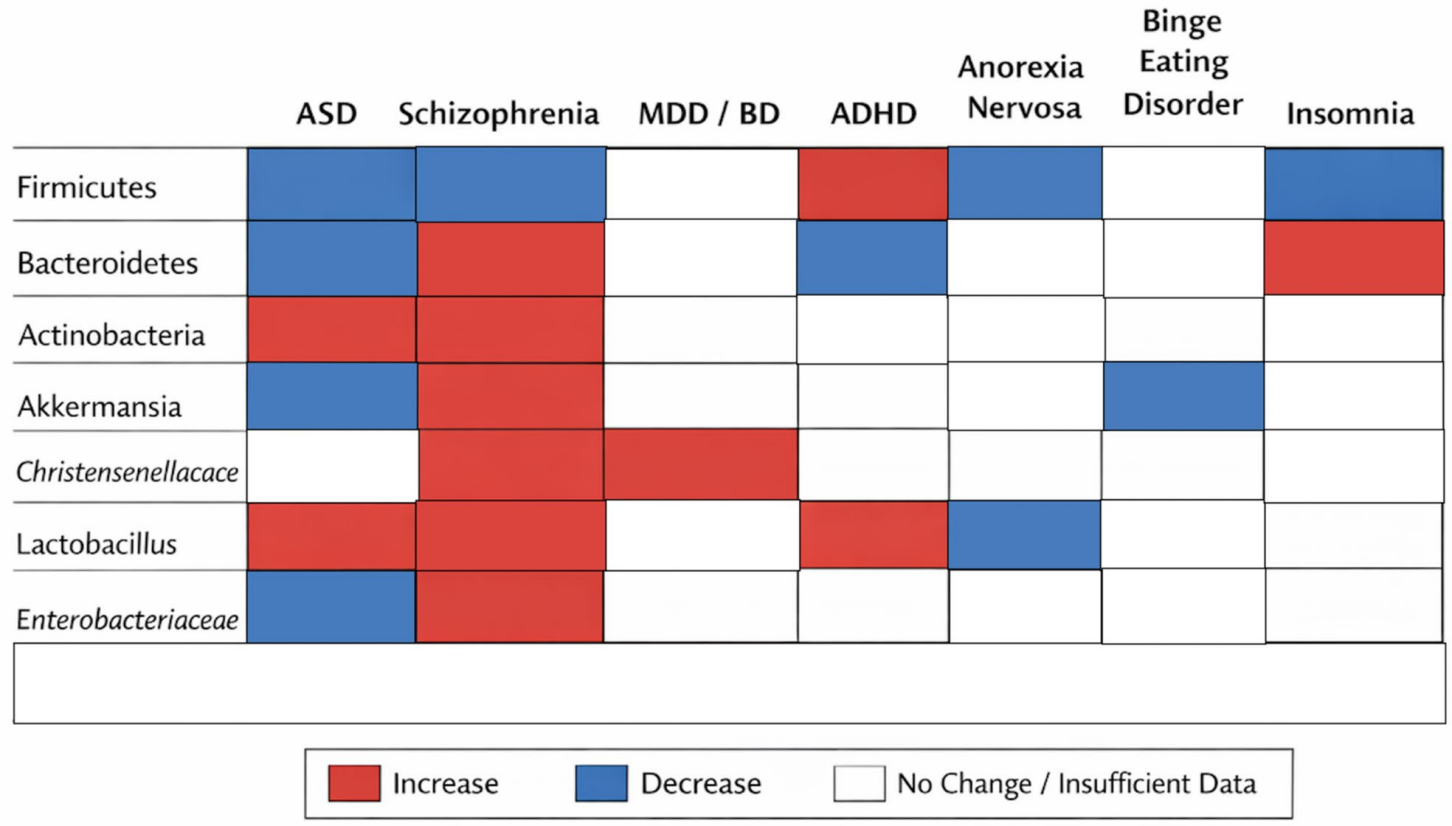
Disorder-wise heatmap of gut microbial alterations across psychiatric conditions. The heatmap summarizes consistent directional changes in key microbial taxa across major psychiatric disorders. Colors indicate the predominant direction of reported change relative to healthy controls (red, increase; blue, decrease; white, inconsistent or insufficient data). The figure highlights both transdiagnostic patterns and disorder-specific microbial signatures derived from the systematic review.

Figure 2, Table 2 showcases a summary of the relevant changes in IM presented as percentage of affected patients and common patterns of change or consequences of the IM change. Table 3 showcases the potential diagnostic implication of taxa alteration in relevant psychiatric conditions included.

**Table 2:** Summary of gut microbiome findings by psychiatric disorder.

Psychiatric disorder	Key microbial changes	Common patterns
Autism spectrum disorder (ASD)	↓ Firmicutes (4.79%) ↓ Bacteroidetes (3.29%) ↑ Actinobacteria (2.87%) ↑ Bifidobacteriaceae (5.86%) ↓ Clostridiaceae (1.97%) ↑ Eggerthellaceae (5.50%) ↓ Sutterella (3.11%)	Reduced diversity, altered SCFA producers, imbalance in key phyla
Mood Disorders (MDD, BD, GAD)	↑ Christensenellaceae (18.10%) ↓ Ruminococcaceae (2.00%) ↑ Faecalibacterium (2.25%) ↑ Flavonifractor (18.10%) ↓ Clostridium XI (6.01%)	Inflammation, barrier dysfunction, SCFA-related bacteria affected
Schizophrenia	↑ Lachnospiraceae (11%) ↑ Christensenellaceae (28%) ↑ Enterobacteriaceae (28%) ↓ Turicibacteraceae (28%) ↓ Pasteurellaceae (28%) ↓ Akkermansia (9%) ↑ Succinivibrio (33%)	Neuroinflammatory and permeability pathways, proinflammatory taxa enriched
Attention deficit hyperactivity disorder (ADHD)	↑ Firmicutes (49.8%) ↓ Bacteroidetes (56.63%),	Imbalanced metabolic/immune gut signaling, reduced diversity. Altered SCFA-producing pathways, linked to inflammation and neurotransmission
Eating disorders (anorexia, binge eating)	*Anorexia:* ↓ Firmicutes (42.7%) ↓ Lactobacillus (48.5%) ↓ Clostridium (47.4%) ↑ Methanobrevibacter (41.5%) *Binge eating:* ↓ Akkermansia (100%) ↑ Megamonas (50.97%)	Severe dysbiosis linked to nutritional deficiency and metabolic shifts
Other disorders (PTSD, sleep, sexual)	*Sleep (insomnia):* ↓ Firmicutes (100%) ↓ Proteobacteria (100%) ↑ Bacteroidetes (100%) *RBD:* ↑ Akkermansia (100%) *Sexual:* ↑ Bifidobacterium (100%) ↑ Lactobacillus (100%) *PTSD:* ↓ Actinobacteria (100%) ↓ Lentisphaerae (100%) ↓ Verrucomicrobia (100%)	Sleep/circadian rhythm and stress-related microbial disruption

ASD, autism spectrum disorder; MDD, major depressive disorder; BD, bipolar disorder; GAD, generalized anxiety disorder; ADHD, attention deficit hyperactivity disorder; PTSD, post-traumatic stress disorder; SCFA, short-chain fatty acids. Arrows indicate direction of change in relative abundance compared with healthy controls (↑ increase, ↓ decrease).

**Table 3:** Summary of disorder-specific microbial taxa alterations and their potential diagnostic implications across major psychiatric conditions.

Disorder	Taxa alteration	Diagnostic implication
Autism spectrum disorder	↓ Firmicutes, ↑ Eggerthellaceae, ↑ Bifidobacteriaceae	Potential for early identification via decreased Firmicutes and increased Eggerthellaceae; candidate for classifier models
Major depressive disorder	↑ Christensenellaceae, ↓ Ruminococcaceae	Discriminative feature in pilot machine learning diagnostic studies
Schizophrenia	↑ Enterobacteriaceae, ↓ Akkermansia, ↑ Lactobacillus	Potential biomarker panel includes Enterobacteriaceae and Akkermansia; supports schizophrenia-specific classifiers
Bipolar disorder	↓ Faecalibacterium, ↑ Bacteroides	May contribute to subtype differentiation using microbiome data
Anorexia nervosa	↓ Lactobacillus, ↓ Firmicutes	Reflects severe malnutrition-associated dysbiosis
Binge eating disorder	↓ Akkermansia (100%)	Complete loss of Akkermansia may serve as a disorder-specific microbial marker
ADHD	↑ Firmicutes, ↓ Bacteroidetes	Imbalance in core phyla could assist in differential diagnosis with other neurodevelopmental disorders

Findings are based on consistent trends reported in the literature and reflect candidate microbial biomarkers for future diagnostic tools. Taxa alterations represent candidate or putative biomarkers based on recurring patterns in the literature and do not imply validated diagnostic markers.

#### 3.3.1 Autism spectrum disorder (ASD)

In the study of various microbial groups concerning patients with autism spectrum disorder (ASD) compared to healthy controls, we included a total of 1,269 participants with ASD; IM changes were observed at the phylum, family and genus levels (Averina et al., 2020; Bojović et al., 2020; Cao et al., 2021; Carissimi et al., 2019; Angelis et al., 2013; Finegold et al., 2010; Gondalia et al., 2012; Grimaldi et al., 2018; Hua et al., 2020; Inoue et al., 2016; Kang et al., 2013, 2017, 2018, 2019; Kong et al., 2019; Kushak et al., 2017; Li et al., 2019; Liu et al., 2017; Liu S. et al., 2019; Luna et al., 2017; Ma et al., 2019; Plaza-Díaz et al., 2019; Shaaban et al., 2018; Son et al., 2015; Strati et al., 2017; Sun et al., 2019; Tomova et al., 2020; Wang et al., 2019a,b; Williams et al., 2011, 2012; Zhai et al., 2019; Zhang et al., 2018; Zhang M. et al., 2020; Zou et al., 2021; Zurita et al., 2020; Pulikkan et al., 2018).

When looking at the phylum level, a 4.79% decrease of *Firmicutes* was reported; this decrease was constant throughout the included studies, with no other articles reporting an increase in this phylum (Angelis et al., 2013; Finegold et al., 2010; Gondalia et al., 2012; Liu S. et al., 2019; Strati et al., 2017; Zhai et al., 2019). On the other hand, the *Actinobacteria* phylum showed a 2.87% increase, with no recorded of decrease (Plaza-Díaz et al., 2019; Wang et al., 2019b). Finally, for the *Bacteroidetes* phylum, a 3.29% decrease was observed, with no data indicating an increase (Williams et al., 2011).

When analysing microbial families, the results were more detailed. The *Prevotella*ceae family showed a 3.11% decrease, while also experiencing a 1.07% increase in other studies (Kang et al., 2013, 2018). In the case of *Bacteroidaceae*, there was a 2.69% decrease reported in some studies (Cao et al., 2021; Williams et al., 2011), but a more significant increase reaching 8.90% reported in others (Kushak et al., 2017; Zhang et al., 2018). The *Peptococcaceae* family showed a 2.10% increase, with no decrease reported (Bojović et al., 2020). In contrast, several bacterial families exhibited only decreases, such as *Enterobacteriaceae* (↓ 1.79%) (Carissimi et al., 2019; Li et al., 2019), *Clostridiaceae* (↓ 1.97%) (Liu S. et al., 2019), *Acidaminococcaceae* (↓ 2.69%) (Ma et al., 2019), *Ruminococcaceae* (↓ 0.53%) (Hua et al., 2020; Inoue et al., 2016), *Aerococcaceae* (↓ 3.77%) (Tomova et al., 2020), and *Odoribacteriaceae* (↓ 1.19%) (Kong et al., 2019). Similarly, other families like *Lactobacillaceae* (↑ 3.53%) (Zurita et al., 2020; Pulikkan et al., 2018), *Bifidobacteraceae* (↑ 5.86%) (Grimaldi et al., 2018; Kang et al., 2017; Shaaban et al., 2018), *Veillonella*ceae (↑ 3.53%) (Zhang M. et al., 2020), and *Eggerthellaceae* (↑ 5.50%) (Liu et al., 2017) only showed increases.

At the genus level, several microbial genera showed notable increases in autistic patients. For example, *Barnesiella* exhibited a 2.1% increase, while *Parabacteroides* saw a similar rise of 2.15% (Averina et al., 2020; Hua et al., 2020). *Agathobacter* and *Faecalibacterium* demonstrated higher increases at 7.18 and 14.30%, respectively, though *Faecalibacterium* also showed a 0.35% decrease in certain cases (Cao et al., 2021; Hua et al., 2020; Inoue et al., 2016). *Blautia* increased by 0.35%, and *Lachnoclostridium* by 2.69% (Inoue et al., 2016; Ma et al., 2019). Increases were also observed in *Prevotella* (4.67%), *Lactobacillus* (4.67%), and *Veillonella* (2.09%) (Zhai et al., 2019; Zhang et al., 2018). Conversely, some genera exhibited decreases in autistic individuals; *Clostridium* levels dropped by 11.67%, while *Cyanobacterium* and *Suterella* showed reductions of 3.53 and 3.11%, respectively (Luna et al., 2017; Son et al., 2015; Williams et al., 2012). Other genera such as *Colinsella*, *Corynebacterium*, *Dorea*, and *Saccharomyces* all exhibited a 4.67% decrease (Zhai et al., 2019; Zou et al., 2021), together with decreases in *Odoribacter* and *Butyrucumonas* (2.09%) (Zhang et al., 2018). *Aspergillus* levels also declined by 1.73%, and *Akkermansia* showed a 1.49% decrease (Zou et al., 2021; Zurita et al., 2020). Additionally, some genera presented both increases and decreases depending on the group of individuals studied. For instance, *Faecalibacterium* increased by 14.30% in some cases but decreased by 0.35% in others (Hua et al., 2020). *Streptococcus* showed a 3.53% decrease in some individuals, while a 2.09% increase was noted in others (Zhang et al., 2018). Similarly, *Bacteroides* increased by 5.50% but also showed a 1.49% decrease, and *Coprococcus* increased by 4.67% while also exhibiting a 1.49% decrease in other instances (Wang et al., 2019a).

#### 3.3.2 Mood disorders

Concerning mood disorders, including major depressive disorder, anxiety disorder, and bipolar disorder we included a total of 768 participants compared against healthy controls; the results show an important difference between patients diagnosed with a mood disorder with regards to the microbial composition (Aizawa et al., 2016; Chen et al., 2018, 2019, 2020; Chung et al., 2019; Coello et al., 2021; Evans et al., 2017; Huang et al., 2018; Jiang et al., 2015; Jiang H. -Y. et al., 2018; Lai et al., 2021; Lin et al., 2017; Lu et al., 2019; Mason et al., 2020; Naseribafrouei et al., 2014; Painold et al., 2019; Rong et al., 2019; Vinberg et al., 2019).

Reported changes in the microbiota’s phyla include an increase in *Firmicutes* of 11.15% in some studies (Lin et al., 2017; Rong et al., 2019) and a 31.43% decrease in others (Mason et al., 2020; Naseribafrouei et al., 2014; Vinberg et al., 2019). Those regarding *Bacteroidetes* showed a 10.64% increase and a 7.58% decrease (Chen et al., 2018, 2019; Chung et al., 2019; Naseribafrouei et al., 2014). Whereas *Actinobacteria* studies reported an 11.21% increase and a 5.20% decrease; and *Bacillota* studies presented only a 11.0% increase (Aizawa et al., 2016; Chen et al., 2018, 2019, 2020; Chung et al., 2019; Coello et al., 2021; Evans et al., 2017; Huang et al., 2018; Jiang et al., 2015; Jiang H. -Y. et al., 2018; Lai et al., 2021; Lin et al., 2017; Lu et al., 2019; Mason et al., 2020; Naseribafrouei et al., 2014; Painold et al., 2019; Rong et al., 2019; Vinberg et al., 2019).

When looking at specific bacterial families, individuals with mood disorders showed a notable 18.10% increase in the *Christensenellaceae* family and a slight 2.00% increase in the *Coriobacteriaceae* family; in contrast, a 2.00% decrease in the *Ruminococcaceae* family was also reported (Aizawa et al., 2016; Chen et al., 2018, 2019, 2020; Chung et al., 2019; Coello et al., 2021; Evans et al., 2017; Huang et al., 2018; Jiang et al., 2015; Jiang H. -Y. et al., 2018; Lai et al., 2021; Lin et al., 2017; Lu et al., 2019; Mason et al., 2020; Naseribafrouei et al., 2014; Painold et al., 2019; Rong et al., 2019; Vinberg et al., 2019).

Lastly, changes in microbiota genera showed, in general, an increase in all bacteria except in the *Clostridium XI* indicating a 6.01% decrease (Aizawa et al., 2016; Chen et al., 2018, 2019, 2020; Chung et al., 2019; Coello et al., 2021; Evans et al., 2017; Huang et al., 2018; Jiang et al., 2015; Jiang H. -Y. et al., 2018; Lai et al., 2021; Lin et al., 2017; Lu et al., 2019; Mason et al., 2020; Naseribafrouei et al., 2014; Painold et al., 2019; Rong et al., 2019; Vinberg et al., 2019). Meanwhile, *Flavinofractor* (18.1%), *Faecalibacterium* (2.25%), *Bacteroides* (6.07%), *Enterobacter*, *Bifidobacterium* (2.25%), *Provotella* (6.01%), *Klebsiella* (3.76%), and *Streptococcus* (3.76%) showed an increase in all cases (Aizawa et al., 2016; Chen et al., 2018, 2019, 2020; Chung et al., 2019; Coello et al., 2021; Evans et al., 2017; Huang et al., 2018; Jiang et al., 2015; Jiang H. -Y. et al., 2018; Lai et al., 2021; Lin et al., 2017; Lu et al., 2019; Mason et al., 2020; Naseribafrouei et al., 2014; Painold et al., 2019; Rong et al., 2019; Vinberg et al., 2019).

#### 3.3.3 Eating disorders

Eating disorders can profoundly impact an individual’s health, not just psychologically but also through significant physical changes, including alterations in their gut microbiome. Two significant eating disorders with notable microbial implications are anorexia nervosa and binge eating; we included a total of 146 participants suffering from eating disorders (Armougom et al., 2009; Borgo et al., 2017; Dong et al., 2020; Hanachi et al., 2019; Kleiman et al., 2015; Leyrolle et al., 2021; Morita et al., 2015).

When looking at anorexia nervosa, several key findings emerged regarding changes in gut microbiota. There was an important decrease in the abundance of several bacterial phyla and genera (Armougom et al., 2009; Borgo et al., 2017; Dong et al., 2020; Hanachi et al., 2019; Kleiman et al., 2015; Leyrolle et al., 2021; Morita et al., 2015). Specifically, the phylum *Firmicutes* was reduced by 42.7%; and regarding the genus, *Lactobacillus* was reduced by 48.5%, *Roseburia* by 42.7%, and *Clostridium* by 47.4% (Armougom et al., 2009; Borgo et al., 2017; Dong et al., 2020; Hanachi et al., 2019; Kleiman et al., 2015; Leyrolle et al., 2021; Morita et al., 2015). These decreases in microbial diversity could be associated with the nutritional deficiencies and metabolic alterations often seen in individuals with anorexia nervosa (Armougom et al., 2009; Kleiman et al., 2015). Conversely, an increase in the genus *Methanobrevibacter*, (41.5%) was also detected (Armougom et al., 2009; Kleiman et al., 2015). This shift in microbial composition may have implications for understanding the complex interactions between diet, gut microbiota, and eating disorders (Borgo et al., 2017; Hanachi et al., 2019; Morita et al., 2015).

Patients with binge eating disorder showed a decrease of the genus *Akkermansia*, with a reduction in 100% of participants (Leyrolle et al., 2021). This is particularly interesting given the role of *Akkermansia* in metabolic health and its potential protective effects against obesity and related disorders. On the other hand, the genus *Megamonas* showed an increase in 50.97% of participants (Dong et al., 2020). These microbial shifts could provide insights into the metabolic and behavioral changes associated with binge eating disorder (Dong et al., 2020; Leyrolle et al., 2021).

#### 3.3.4 Attention deficit hyperactivity disorder

Attention deficit hyperactivity disorder (ADHD) is a complex neurodevelopmental condition characterized by persistent patterns of inattention, hyperactivity, and impulsivity; recent research has explored the link between ADHD and gut microbiome composition, revealing significant microbial shifts that may contribute to the disorder’s manifestations. We included a total of 107 participants diagnosed with ADHD in our review (Aarts et al., 2017; Pärtty et al., 2015; Jiang H. et al., 2018; Prehn-Kristensen et al., 2018; Wan et al., 2020).

Our analysis highlighted an important increase in the abundance of the phylum *Firmicutes*, which rose in 49.8% of participants; these are a diverse group of bacteria involved in various metabolism and immune function aspects (Aarts et al., 2017; Pärtty et al., 2015; Jiang H. et al., 2018; Prehn-Kristensen et al., 2018; Wan et al., 2020). The increased presence of *Firmicutes* could be related to altered metabolic processes and inflammation, often associated with ADHD. Conversely, a decrease in the phylum *Bacteroidetes*, with a reduction in 56.63% of participants was also detected (Aarts et al., 2017; Pärtty et al., 2015; Jiang H. et al., 2018; Prehn-Kristensen et al., 2018; Wan et al., 2020). *Bacteroidetes* are crucial for breaking down complex carbohydrates and maintaining gut health; a decrease in this phylum could impair metabolic functions and affect the gut’s ability to regulate inflammation and nutrient absorption, potentially influencing ADHD symptoms (Jiang H. et al., 2018). This imbalance between *Firmicutes* and *Bacteroidetes* may contribute to the dysregulation of neurotransmitter systems and inflammatory pathways, which are implicated in the pathophysiology of ADHD (Wan et al., 2020).

#### 3.3.5 Schizophrenia

In examining the composition of the gut microbiota between patients with schizophrenia and healthy controls, we included a total of 323 participants with the disease (Li et al., 2020; Ma et al., 2020; Nguyen et al., 2019; Nguyen et al., 2021; Pan et al., 2020; Pełka-Wysiecka et al., 2019; Shen et al., 2018; Yuan et al., 2018; Zhang X. et al., 2020).

When looking at the phylum level, patients with schizophrenia have an increase in *Proteobacteria* in 23% of patients. Additionally, *Firmicutes* and *Bacteroidetes* showed an increase in 7% of patients; while *Actinobacteria* was increased in 25% of patients (Li et al., 2020; Ma et al., 2020; Nguyen et al., 2019; Nguyen et al., 2021; Pan et al., 2020; Pełka-Wysiecka et al., 2019; Shen et al., 2018; Yuan et al., 2018; Zhang X. et al., 2020). On the other hand, the prevalence of *Proteobacteria* was found to be markedly reduced in 6% of patients with schizophrenia, while the presence of *Firmicutes* was also lowered in 19% of these patients (Li et al., 2020; Nguyen et al., 2019; Pan et al., 2020; Shen et al., 2018; Zhang X. et al., 2020).

We also found interesting differences in specific bacterial families in patients with schizophrenia. For instance, our data revealed a notable increase in the abundance of the *Lachnospiraceae*, *Christensenellaceae*, and *Enterobacteriaceae* families in 11, 28, and 28% of patients, respectively (Li et al., 2020; Ma et al., 2020; Nguyen et al., 2019; Nguyen et al., 2021; Pan et al., 2020; Pełka-Wysiecka et al., 2019; Shen et al., 2018; Yuan et al., 2018; Zhang X. et al., 2020). In contrast, we also found a decrease in the *Turicibacteraceae* (28%), *Pasteurellaceae* (28%), and *Lachnospiraceae* (2%) families (Li et al., 2020; Ma et al., 2020; Nguyen et al., 2019; Nguyen et al., 2021; Pan et al., 2020; Pełka-Wysiecka et al., 2019; Shen et al., 2018; Yuan et al., 2018; Zhang X. et al., 2020).

When analysing patients with schizophrenia at the genus level, *Succinivibrio* was identified as the most prevalent, being increased in 33% of the patients (Li et al., 2020; Ma et al., 2020; Nguyen et al., 2019; Nguyen et al., 2021; Pan et al., 2020; Pełka-Wysiecka et al., 2019; Shen et al., 2018; Yuan et al., 2018; Zhang X. et al., 2020). The genus *Clostridium* emerged as a significant component of the microbiome, being prevalent in 28% of the patients (Li et al., 2020; Ma et al., 2020; Nguyen et al., 2019, 2021; Pan et al., 2020; Pełka-Wysiecka et al., 2019; Shen et al., 2018; Yuan et al., 2018; Zhang X. et al., 2020). Other genera such as *Mogibacterium*, *Corynebacterium*, *Eubacterium*, and *Lactobacillus* were also prevalent and increased in 19% of participants; *Megasphera*, *Collisinella*, *Klebsiella*, and *Methanobrevibacter* were also increased in 14% of the individuals (Li et al., 2020; Ma et al., 2020; Nguyen et al., 2019, 2021; Pan et al., 2020; Pełka-Wysiecka et al., 2019; Shen et al., 2018; Yuan et al., 2018; Zhang X. et al., 2020). In similar fashion, other genera showed a modest increase with *Anaerococcus* being increased in 6% and *Bacteroides* and *Blautia* in 5% of patients with schizophrenia (Li et al., 2020; Nguyen et al., 2019; Pełka-Wysiecka et al., 2019; Shen et al., 2018; Yuan et al., 2018). In contrast, the genera *Clostridium*, *Haemophilus*, and *Sutterella* show a uniform reduction, each being lowered in 6% of patients (Li et al., 2020; Ma et al., 2020; Nguyen et al., 2019, 2021; Pan et al., 2020; Pełka-Wysiecka et al., 2019; Shen et al., 2018; Yuan et al., 2018; Zhang X. et al., 2020). More important reductions are observed with *Blautia*, *Coprococcus*, and *Roseburia*, each diminished in 14% of the patients; *Lactobacillus*, *Escherichia*, and *Bifidobacteria* in 9% of patients; and *Faecalibacterium*, *Adlercreutzia*, and *Anaerostipes* in 21, 19, and 19% of patients, respectively (Li et al., 2020; Ma et al., 2020; Nguyen et al., 2019, 2021; Pan et al., 2020; Pełka-Wysiecka et al., 2019; Shen et al., 2018; Yuan et al., 2018; Zhang X. et al., 2020).

#### 3.3.6 Other disorders

We found evidence regarding changes of IM in other psychiatric disorders including sleep disorders (*n* = 36), hypoactive sexual disorder (*n* = 24), and post-traumatic stress disorder (PTSD) (*n* = 18) for a total of 78 participants (Hemmings et al., 2017; Li et al., 2021; Liu B. et al., 2019; Nishiwaki et al., 2020a).

Sleep disorders can profoundly affect an individual’s quality of life and overall health. Recent studies have shed light on the possible relationships between gut microbiome and sleep disorders; here we found information about two sleep disorders with distinct microbial alterations (insomnia disorder and idiopathic REM sleep behavior disorder) (Liu B. et al., 2019; Nishiwaki et al., 2020a). Insomnia disorder, characterized by persistent difficulty in falling or staying asleep, was linked to a decrease of the genera *Firmicutes* and *Proteobacteria* in 100% of the patients in the included study (Liu B. et al., 2019). These genera are integral to maintaining gut health and metabolic balance and their disruption may contribute to disruptions in sleep patterns through mechanisms related to inflammation and metabolic disturbances (Liu B. et al., 2019). Conversely, the same study found a complete increase of the *Bacteroidetes* genus in 100% of individuals with insomnia (Liu B. et al., 2019). In contrast, idiopathic REM sleep behavior disorder (RBD) is characterized by the abnormal enactment of dreams during REM sleep, often leading to disruptive and potentially dangerous behaviors. In a study of 163 participants (26 with idiopathic RBD and 137 controls) the genus and family *Akkermansia* showed a dramatic increase in 100% of RBD participants (Nishiwaki et al., 2020a). *Akkermansia* is known for its role in maintaining gut barrier function and modulating inflammation and its increase may be related to the disorder’s pathophysiology, potentially influencing sleep patterns and behavior through effects on the gut-brain axis (Liu B. et al., 2019).

Hypoactive sexual disorder, characterized by a reduced interest in sexual activity; we only found one study reporting an increase in the genera *Bifidobacterium* and *Lactobacillus* in 100% of participants (Li et al., 2021). Certainly, *Bifidobacterium* is involved in the production of beneficial metabolites and modulation of immune responses, while *Lactobacillus* helps maintain a healthy gut environment and support vaginal health (Li et al., 2021). The increase in these genera might reflect an adaptive response to the disorder or indicate a shift in the microbiome that impacts sexual health. Further research is needed to understand the precise mechanisms by which these microbial changes influence sexual function and to explore potential therapeutic approaches.

Finally, regarding individuals with post-traumatic stress disorder (PTSD), we only found one study reporting a complete decrease of the phyla *Actinobacteria*, *Lentisphaerae*, and *Verrucomicrobia* in 100% of the studied individuals (Hemmings et al., 2017). These phyla play important roles in maintaining gut homeostasis and modulating immune responses (Hemmings et al., 2017).

## 4 Discussion

Our systematic review contributes to the novel area of investigation of the gut microbiome and its relationship with different mental diseases by compiling and analysing the current body of evidence regarding gut microbial changes in psychiatric disease and their potential as candidate diagnostic biomarkers. This synthesis underscores the burgeoning interest in deciphering how the gut microbiota, the complex community of microorganisms residing in the human gastrointestinal tract, impacts mental health. Moreover, our review emphasizes what are the predominant phylum, family and genus of bacteria altered in specific mental disorders like autism spectrum disorder, schizophrenia, mood disorders, and others. The findings of this systematic review reveal consistent and significant alterations in the intestinal microbiome (IM) composition, emphasizing the critical role of the microbiome-gut-brain axis in mental health; these findings underscore the diagnostic potential of microbial signatures and their role in future biomarker-guided stratification of psychiatric conditions.

### 4.1 Diagnostic implications and clinical translation

The findings of this review reveal reproducible, disorder-specific patterns of gut microbial dysbiosis that may have diagnostic utility in psychiatry. Notably, decreased Firmicutes in ASD, increased Enterobacteriaceae in schizophrenia, and complete loss of Akkermansia in eating disorders highlight taxa that may distinguish clinical phenotypes. Although larger absolute reductions in Firmicutes were observed in conditions such as anorexia nervosa and insomnia, these changes are likely driven by acute nutritional deprivation or disorder-specific physiological states rather than stable, disorder-specific microbial signatures. In contrast, the reduction of Firmicutes in autism spectrum disorder has been consistently reported across multiple independent cohorts and age groups, supporting its potential relevance as a reproducible and diagnostically informative feature rather than a secondary metabolic consequence. These microbial signatures offer promise as non-invasive, adjunctive tools for early or differential diagnosis. Certainly, the application of microbial panels, comprising combinations of discriminative taxa, has already been explored in pilot studies. For instance, classifiers trained on faecal microbiota data have achieved moderate-to-high diagnostic accuracy in distinguishing patients with major depressive disorder, schizophrenia, and ASD from healthy controls. Machine learning-based algorithms, using genus- or family-level microbiota features, have shown preliminary success in differentiating disease subtypes and predicting symptom severity. These computational approaches represent a scalable framework for microbiome-based diagnostic development. Furthermore, biomarker assays targeting microbial metabolites or microbial DNA are being developed as potential clinical tests. For example, altered levels of short-chain fatty acids, trimethylamine-N-oxide (TMAO), or lipopolysaccharide-producing bacteria may reflect underlying pathophysiology and could be incorporated into multimodal diagnostic workflows.

However, current evidence remains preliminary. Variation in study design, sampling techniques, and bioinformatics pipelines limits generalisability. For microbiome signatures to become clinically actionable diagnostic tools, prospective longitudinal studies with standardised methods and independent validation cohorts are needed. Nevertheless, our findings provide a foundation for the development of potential microbiome-informed diagnostic frameworks in psychiatry. However, it is important to distinguish between disorder-specific microbial signatures and transdiagnostic patterns shared across multiple psychiatric conditions. Several taxa highlighted in this review, such as alterations in the Firmicutes–Bacteroidetes ratio or changes in Christensenellaceae, appear across multiple diagnostic categories and are therefore more likely to reflect shared metabolic, inflammatory, or lifestyle-related factors rather than disorder-specific markers. In contrast, certain microbial alterations, such as the complete loss of Akkermansia in binge eating disorder or reproducible reductions in Firmicutes in autism spectrum disorder, may represent more diagnosis-informative candidate signatures if properly replicated in future studies.

### 4.2 Microbial composition differences

Across disorders, dysbiosis appears to be characterized by a general imbalance in the phyla *Firmicutes*, *Bacteroidetes*, and *Actinobacteria*, along with disorder-specific fluctuations at the family and genus levels. These shifts underscore a shared microbial signature associated with psychiatric symptomatology, while also pointing to unique microbial alterations per disorder, suggesting a multifactorial and diagnosis-specific microbiota-psychopathology relationship.

Across disorders, dysbiosis was most consistently characterized by a disruption of the Firmicutes–Bacteroidetes balance rather than a uniform directional change (Aarts et al., 2017; Aizawa et al., 2016; Armougom et al., 2009; Averina et al., 2020; Bojović et al., 2020; Borgo et al., 2017; Cao et al., 2021; Carissimi et al., 2019; Chen et al., 2018, 2019, 2020; Chung et al., 2019; Coello et al., 2021; Angelis et al., 2013; Dong et al., 2020; Evans et al., 2017; Finegold et al., 2010; Gondalia et al., 2012; Grimaldi et al., 2018; Hanachi et al., 2019; Hemmings et al., 2017; Hua et al., 2020; Huang et al., 2018; Inoue et al., 2016; Pärtty et al., 2015; Jiang et al., 2015; Jiang H. -Y. et al., 2018; Jiang H. et al., 2018; Kang et al., 2013, 2017, 2018, 2019; Kleiman et al., 2015; Kong et al., 2019; Kushak et al., 2017; Lai et al., 2021; Leyrolle et al., 2021; Li et al., 2019, 2021, 2020; Lin et al., 2017; Liu B. et al., 2019; Liu et al., 2017; Liu S. et al., 2019; Lu et al., 2019; Luna et al., 2017; Ma et al., 2019, 2020; Mason et al., 2020; Morita et al., 2015; Naseribafrouei et al., 2014; Nguyen et al., 2019, 2021; Nishiwaki et al., 2020a; Painold et al., 2019; Pan et al., 2020; Pełka-Wysiecka et al., 2019; Plaza-Díaz et al., 2019; Prehn-Kristensen et al., 2018; Rong et al., 2019; Shaaban et al., 2018; Shen et al., 2018; Son et al., 2015; Strati et al., 2017; Sun et al., 2019; Tomova et al., 2020; Vinberg et al., 2019; Wan et al., 2020; Wang et al., 2019a,b; Williams et al., 2011, 2012; Yuan et al., 2018; Zhai et al., 2019; Zhang et al., 2018; Zhang M. et al., 2020; Zhang X. et al., 2020; Zou et al., 2021; Zurita et al., 2020; Pulikkan et al., 2018). While the magnitude and direction of Firmicutes and Bacteroidetes alterations varied by diagnosis, this recurrent imbalance suggests shared perturbations in core metabolic and immunomodulatory pathways across psychiatric conditions. These findings are consistent with the disorder-specific patterns reported in the Results section and Table 2, where both increases and decreases in Firmicutes and Bacteroidetes were observed depending on the psychiatric phenotype. *Firmicutes* are generally associated with butyrate production and intestinal barrier integrity, while *Bacteroidetes* play roles in polysaccharide digestion and immune regulation (Qin et al., 2010; Rinninella et al., 2019); disruption in this ratio has been previously linked with neuroinflammatory processes and altered neurotransmitter production (Kelly et al., 2016; Dinan and Cryan, 2015). Additionally, the increase in *Actinobacteria*, particularly *Bifidobacteria*ceae, in disorders such as ASD and schizophrenia, may represent a compensatory response or reflect underlying dietary and metabolic adaptations (Strati et al., 2017; Zhang et al., 2018; O’Mahony et al., 2009).

When looking at specific psychiatric disorders, patients with ASD consistently exhibited reductions in *Firmicutes* and *Bacteroidetes*, and increases in *Actinobacteria*, *Eggerthellaceae*, and *Bifidobacteria*ceae. Genus-level increases in *Faecalibacterium*, *Prevotella*, and *Parabacteroides*, coupled with marked reductions in *Clostridium* and *Sutterella*, align with studies linking ASD to microbial pathways involved in GABA and serotonin metabolism (Finegold et al., 2010; Luna et al., 2017). Furthermore, reduced microbial diversity and altered short-chain fatty acid (SCF) production, particularly butyrate, may influence neurodevelopmental trajectories and social behavior through epigenetic and neuroimmune pathways (Sharon et al., 2019).

In major depressive disorder and bipolar disorder, shifts in *Christensenellaceae* (↑ 18%) and *Ruminococcaceae* (↓ 2%) were salient. These families have been implicated in gut permeability and pro-inflammatory cytokine production, mechanisms increasingly recognized in depression’s pathophysiology (Mason et al., 2020; Zheng et al., 2016). Elevated levels of *Faecalibacterium* and *Flavonifractor* suggest a compensatory attempt at restoring gut-brain homeostasis; however, reductions in *Clostridium* XI (↓ 6.01%) may indicate a persistent dysbiotic state unable to support proper neuromodulatory signaling (Painold et al., 2019; Evans et al., 2017).

Individuals with schizophrenia exhibited elevated *Enterobacteriaceae* and *Christensenellaceae*, along with reductions in *Turicibacteraceae* and *Pasteurellaceae*. These alterations points toward the possibility of increased gut permeability, systemic inflammation, and microbial-derived metabolites such as D-lactic acid and lipopolysaccharides, which have been shown to breach the blood–brain barrier and modulate microglial activation (Pełka-Wysiecka et al., 2019; Fung et al., 2017; McGuinness et al., 2022). Genera such as *Succinivibrio* and *Clostridium* were frequently increased, echoing earlier reports of their role in dopaminergic modulation (Xu et al., 2020).

Anorexia nervosa was characterized by sharp declines in *Lactobacillus*, *Clostridium*, and *Roseburia*, reflecting a state of nutrient deficiency and intestinal inflammation (Armougom et al., 2009; Hanachi et al., 2019; Di Lodovico et al., 2021). In contrast, *Methanobrevibacter* was consistently elevated, suggesting altered fermentation and methane metabolism, possibly contributing to constipation and delayed intestinal transit (Xu et al., 2022). Binge eating disorder revealed a striking 100% decrease in *Akkermansia*, a mucin-degrading genus critical for gut barrier function and metabolic health (Dao et al., 2016).

ADHD presented a unique profile of *Firmicutes* elevation and *Bacteroidetes* reduction. This mirrors prior work suggesting that *Firmicutes* dominance may modulate impulsivity and hyperactivity through altered SCFA signalling and dopamine turnover (Prehn-Kristensen et al., 2018). Additionally, changes in *Bacteroidetes*, noted for their role in polysaccharide degradation, may affect systemic energy availability and inflammatory tone (Wang et al., 2020).

Finally, the reviewed studies on PTSD and sleep disorders pointed to phylum-level losses in *Verrucomicrobia*, *Lentisphaerae*, and *Actinobacteria*, and genus-level gains in *Akkermansia* (RBD) and *Bacteroides* (insomnia). These changes may reflect altered circadian rhythms and stress-related neuroendocrine dysfunctions influencing microbial ecology (Nishiwaki et al., 2020b; Lin et al., 2024).

### 4.3 Future directions and limitations

While causality remains elusive, the associations presented in our review support the growing rationale for the link between the microbiota and psychiatric disorders and the future potential of therapies directed at microbiota in psychiatry and the use of these dysbiosis patterns as potential biomarkers; something that still requires further high-quality investigation, with standardized protocols and different types of populations. Interventions such as probiotic supplementation, prebiotics, and faecal microbiota transplantation are currently under investigation, particularly in depression and ASD (Wallace and Milev, 2017; Liu R. T. et al., 2019; Slykerman et al., 2018). Early trials suggest improvements in mood, social behavior, and inflammation markers, though heterogeneity in strain specificity, delivery method, and patient phenotype remains a challenge (Kumar et al., 2024). The existing literature, while promising, underscores the necessity for further, more nuanced research to unravel the complexities of the microbiome’s contributions to mental health. This includes elucidating the mechanisms through which the microbiome influences brain function and mental well-being, understanding how interventions can be tailor-made to harness the microbiome for mental health benefits, and identifying specific microbial signatures that could predict response to treatment or the course of mental illnesses.

Advancing the diagnostic utility of gut microbiome research in psychiatry requires a systematic shift toward biomarker development, validation, and clinical translation. While current findings reveal disorder-specific microbial alterations, such as ↓ Firmicutes in ASD or ↑ Enterobacteriaceae in schizophrenia, these must now be leveraged to build clinically useful tools. First, there is a need for large-scale, longitudinal studies with harmonized protocols for sample collection, sequencing, and analysis. Diagnostic performance metrics (e.g., sensitivity, specificity, AUC) of candidate microbial biomarkers should be reported consistently. Multi-site cohorts with diverse geographic and ethnic representation will be essential to confirm generalisability. Second, the development of diagnostic classifiers based on machine learning algorithms trained on microbiota profiles represents a promising avenue. Early pilot studies suggest that microbial data alone, or in combination with symptom ratings and metabolomics, may predict psychiatric diagnoses with moderate-to-high accuracy. Future work should prioritise the refinement and external validation of these models. Third, integration with multi-omics platforms (metabolomics, proteomics, and host genomics) could enhance diagnostic resolution. For instance, combining gut microbiota data with microbial-derived metabolites such as short-chain fatty acids or neurotransmitter precursors may yield composite biomarkers with greater discriminative power. Finally, regulatory, ethical, and practical considerations must be addressed. Establishing standardised thresholds, reproducible pipelines, and clinical-grade assays will be crucial for transitioning microbiome-based diagnostics from research to psychiatric practice. Interdisciplinary collaboration across psychiatry, microbiology, data science, and regulatory science will be essential to realise this potential.

Interpreting the results of this systematic review should be accompanied by understanding its potential limitations. First, the predominance of cross-sectional designs in the included research restricts the capacity to properly establish causal links between gut microbiota changes and psychiatric diseases. Still rare are longitudinal and interventional investigations, which limit understanding of the temporal dynamics of microbiome changes in respect to disease development, progression, and treatment response. The moderate overall methodological quality of the included studies further constrains diagnostic inference. Given that many studies exhibited limitations in participant selection, confounder control, and microbiome assessment, proposed microbial signatures should be considered exploratory and hypothesis-generating rather than diagnostically actionable. This underscores the need for higher-quality, prospectively designed studies before microbiome-based diagnostic tools can be reliably developed. Additionally, the percentage-based summaries reported in this review are descriptive and intended to convey the relative recurrence of microbial alterations across heterogeneous studies. They do not constitute prevalence estimates or effect sizes and should be interpreted cautiously, particularly given variation in study design, sequencing methods, and reporting practices.

Second, substantial methodological heterogeneity is present across included studies. Sequencing approaches varied widely, with some studies employing 16S rRNA gene sequencing and others using shotgun metagenomic sequencing, resulting in differences in taxonomic resolution, sensitivity, and functional inference. In addition, reporting was inconsistent across taxonomic levels, with some studies providing phylum- or family-level data only, while others reported genus- or species-level alterations. This heterogeneity limits direct comparability of microbial findings across studies. Beyond technical variability, multiple clinical and environmental confounders were insufficiently controlled across studies. Psychotropic medication exposure, particularly antipsychotics and antidepressants, is known to independently alter gut microbiota composition and was variably reported or adjusted for. Similarly, body mass index, physical activity, lifestyle factors, and geographic and cultural background differed markedly across cohorts and were often incompletely documented. These variables are well-established determinants of gut microbial structure and likely contributed to inter-study heterogeneity and inconsistent findings. Given this degree of methodological, clinical, and environmental heterogeneity, a formal quantitative meta-analysis was not feasible. The lack of standardized sequencing platforms, inconsistent taxonomic reporting, variable outcome definitions, and insufficient adjustment for key confounders would have rendered pooled effect estimates unreliable and potentially misleading. Consequently, we adopted a structured descriptive synthesis to summarize recurring patterns of microbial alteration while avoiding overinterpretation of heterogeneous data.

Third, neither consistently controlled for nor reported dietary habits, drug use (especially psychiatric medication and antibiotics), nor concomitant medical disorders. The lack of standardizing in these variables presents possible uncontrolled confounding factors that might have affected the detected microbial signatures given their recognized effect on gut microbiota composition.

Fourth, psychiatric diagnosis itself remains clinically diverse despite the inclusion of only studies using DSM-IV, DSM-V, or comparable validated diagnostic methods; symptom severity, disease duration, and treatment history were often insufficiently documented; this lack of detail restricts the discovery of microbiome patterns linked with particular subtypes or phases of psychiatric disease. Certainly, dietary intake represents a major unaddressed confounding factor in the current literature on the gut-brain axis. Gut microbiome composition is highly sensitive to dietary patterns, including macronutrient distribution, fiber intake, consumption of ultra-processed foods, and intake of fermented products. Most studies included in this review did not systematically assess or control for participants’ dietary habits, which likely contributes substantially to inter-study heterogeneity and inconsistent microbial findings across psychiatric disorders. Consequently, some reported microbial alterations may reflect dietary effects rather than disorder-specific pathophysiology. Future studies should incorporate standardized dietary assessments, such as validated food frequency questionnaires, 24-h dietary recalls, or dietary pattern indices, and consider dietary stratification or adjustment in statistical models. Controlled feeding designs or run-in dietary standardization periods may further reduce confounding. Integrating dietary data with microbiome, metabolomic, and clinical phenotyping will be essential to disentangle diet-driven microbial variation from psychiatric disease-related signatures.

Lastly, few research involved different cohorts, therefore restricting the generalizability of results; most studies were carried out in particular regional or ethnic communities. The microbiome is clearly shaped by host genes, environment, and sociocultural elements, so, future research should aim to include more general demographic and geographic representation.

Taken together, these constraints draw attention to the need of standardized methods, bigger and more varied cohorts, and longitudinal multi-omics techniques to better grasp the function of the gut microbiome in psychiatric diseases and its potential for diagnostic and therapeutic innovation.

## 5 Conclusion

This systematic review reveals potential disorder-specific patterns of gut microbial dysbiosis across major psychiatric conditions. Alterations such as ↓ *Firmicutes* in autism spectrum disorder, ↑ *Enterobacteriaceae* in schizophrenia, and complete loss of *Akkermansia* in eating disorders highlight microbial signatures with potential diagnostic relevance that warrant validation in longitudinal and interventional studies. These findings support the emerging role of the gut microbiome not only in the pathophysiology of psychiatric disorders but also as a promising source of non-invasive biomarkers. As the field advances, the integration of microbiota profiling into clinical diagnostics could improve early detection, differential diagnosis, and patient stratification; however, we are far from there yet. Realising this potential will require rigorous validation in large-scale, standardised studies and the development of clinically actionable microbial panels or predictive models. Ultimately, translating gut microbiome signatures into reliable diagnostic tools could transform psychiatric evaluation, offering novel avenues for precision psychiatry and biomarker-guided care.

## Data Availability

The datasets presented in this study can be found in online repositories. The names of the repository/repositories and accession number(s) can be found in the article/Supplementary material.
