# Supplementary material for: Microbial dysbiosis as a diagnostic marker in psychiatric disorders: a systematic review of gut–brain axis disruptions
Source: Front Neurosci. 2026 Feb 3;20:1728473. doi: 10.3389/fnins.2026.1728473 (PMC12909529; doi:10.3389/fnins.2026.1728473)
Supplement: Supplementary file 1 [file Data_Sheet_1.docx]

**Supplementary Materials**

**PubMed (MEDLINE) search strategy:**

(Neuropsychiatric OR Psychiatric OR Neuropsychiatric disease OR Psychiatric disease OR mental disorder OR psychiatric illness OR mental illness OR ("Mental disorders" [MeSH])) AND (Microbiome OR Intestinal microbiome OR gastrointestinal microbiome OR gut microbiome OR intestinal microbiota OR gut microflora OR gastrointestinal microflora OR gut flora OR gastric microbiota OR gastric flora OR enteric bacteria OR ("Gastrointestinal Microbiome"[Mesh])) AND (progression OR prognos* OR prognostic factor OR pathogenesis OR etiology OR risk OR risk factor OR development OR precipitating factors OR protective factors OR causality OR ("causality" [MeSH]) OR ("etiology" [Subheading]) OR ("Prognosis" [MeSH]) OR ("Risk factors" [MeSH]))

**SCOPUS search strategy:**

TITLE-ABS-KEY ((Neuropsychiatric OR Psychiatric OR (Neuropsychiatric disease) OR (Psychiatric disease) OR (mental disorder) OR (psychiatric illness) OR (mental illness)) AND (microbiome OR (Intestinal microbiome) OR (gastrointestinal microbiome) OR (gut microbiome) OR (intestinal microbiota) OR (gut microflora) OR (gastrointestinal microflora) OR (gut flora) OR (gastric microbiota) OR (gastric flora) OR (enteric bacteria)) AND ((progression) OR (prognos*) OR (prognostic factor) OR (pathogenesis) OR (etiology) OR risk OR (risk factor) OR (development) OR (precipitating factors) OR (protective factors) OR (causality)))

**CENTRAL search strategy:**

ID Search Hits

#1 Neuropsychiatric

#2 Psychiatric

#3 Neuropsychiatric disease

#4 psychiatric disease

#5 mental disorders

#6 psychiatric illness

#7 mental illness

#8 MeSH descriptor: [Mental Disorders] explode all trees

#9 #1 OR #2 OR #3 OR #4 OR #5 OR #6 OR #7 OR #8

#10 Intestinal microbiome

#11 gastrointestinal microbiome

#12 gut microbiome

#13 intestinal microbiota

#14 gut microflora

#15 gastrointestinal microflora

#16 gut flora

#17 gastric microbiota

#18 gastric flora

#19 enteric bacteria

#20 MeSH descriptor: [Gastrointestinal Microbiome] explode all trees

#21 #10 OR #11 OR #12 OR #13 OR #14 OR #15 OR #16 OR #17 OR #18 OR #19 OR #20

#22 progression

#23 prognos*

#24 prognostic factors

#25 pathogenesis

#26 etiology

#27 risk

#28 risk factors

#29 development

#30 precipitating factors

#31 protective factors

#32 causality

#33 MeSH descriptor: [Causality] explode all trees

#34 MeSH descriptor: [Prognosis] explode all trees

#35 MeSH descriptor: [Risk Factors] explode all trees

#36 #22 OR #23 OR #24 OR #25 OR #26 OR #27 OR #28 OR #29 OR #30 OR #31 OR #32 OR #33 OR #34 OR #35

#37 #9 AND #21 AND #36

**PsychINFO search strategy:**

(Neuropsychiatric OR Psychiatric OR (Neuropsychiatric disease) OR (Psychiatric disease) OR (mental disorder) OR (psychiatric illness) OR (mental illness)) AND (Microbiome OR (Intestinal microbiome) OR (gastrointestinal microbiome) OR (gut microbiome) OR (intestinal microbiota) OR (gut microflora) OR (gastrointestinal microflora) OR (gut flora) OR (gastric microbiota) OR (gastric flora) OR (enteric bacteria)) AND (progression OR prognos* OR (prognostic factor) OR pathogenesis OR etiology OR risk OR (risk factor) OR development OR (precipitating factor) OR (protective factor) OR causality)
